# Analyses of Mosquito Species Composition, Blood-Feeding Habits and Infection with Insect-Specific Flaviviruses in Two Arid, Pastoralist-Dominated Counties in Kenya

**DOI:** 10.3390/pathogens12070967

**Published:** 2023-07-24

**Authors:** Edwin O. Ogola, Armanda D. S. Bastos, Gilbert Rotich, Anne Kopp, Inga Slothouwer, Dorcus C. A. Omoga, Rosemary Sang, Baldwyn Torto, Sandra Junglen, David P. Tchouassi

**Affiliations:** 1 International Centre of Insect Physiology and Ecology (icipe), Nairobi P.O. Box 30772-00100, Kenyadomoga@icipe.org (D.C.A.O.);; 2 Department of Zoology and Entomology, University of Pretoria, Private Bag 20, Pretoria 0028, South Africa; 3 Institute of Virology, Charité Universitätsmedizin Berlin, Corporate Member of Free University Berlin, Humboldt-University Berlin, and Berlin Institute of Health, Chariteplatz 1, 10117 Berlin, Germany

**Keywords:** arbovirus surveillance, mosquitoes, blood-fed, *Flavivirus*, CuaCua virus, Kenya

## Abstract

Insect-specific flaviviruses (ISFs), although not known to be pathogenic to humans and animals, can modulate the transmission of arboviruses by mosquitoes. In this study, we screened 6665 host-seeking, gravid and blood-fed mosquitoes for infection with flaviviruses and assessed the vertebrate hosts of the blood-fed mosquitoes sampled in Baringo and Kajiado counties; both dryland ecosystem counties in the Kenyan Rift Valley. Sequence fragments of two ISFs were detected. Cuacua virus (CuCuV) was found in three blood-fed *Mansonia* (*Ma.*) *africana*. The genome was sequenced by next-generation sequencing (NGS), confirming 95.8% nucleotide sequence identity to CuCuV detected in *Mansonia* sp. in Mozambique. Sequence fragments of a potential novel ISF showing nucleotide identity of 72% to Aedes flavivirus virus were detected in individual blood-fed *Aedes aegypti*, *Anopheles gambiae* s.l., *Ma. africana* and *Culex* (*Cx.*) *univittatus*, all having fed on human blood. Blood-meal analysis revealed that the collected mosquitoes fed on diverse hosts, primarily humans and livestock, with a minor representation of wild mammals, amphibians and birds. The potential impact of the detected ISFs on arbovirus transmission requires further research.

## 1. Introduction

Arthropod-borne viruses are transmitted by blood-feeding arthropods such as mosquitoes. Among the recent epidemics occurring in the Afrotropical region (Africa south of the Sahara Desert), the majority were caused by arboviruses of the genus *Flavivirus* (family *Flaviviridae*) [1]. This is exemplified by the re-emergence of yellow fever and dengue viruses (YFV and DENV) in Kenya [2,3,4,5], Zika virus (ZIKV) in Angola and Cape Verde [6,7], and West Nile virus (WNV) in Gabon [8]. Apart from these mosquito-borne flaviviruses (MBF), no-known-vector flaviviruses (NKV) have been described, such as Rio Bravo and Modoc viruses which only infect vertebrates [9,10]. Another category of flaviviruses are insect-specific flaviviruses (ISFs), which replicate exclusively in insects and are unable to infect vertebrates. Examples include Kamiti River virus (KRV), Cuacua virus (CuCuV) and Niénokoué virus (NIEV) [11,12,13]. Insect-specific flaviviruses are divided into two phylogenetic groups. The classical ISFs (cISFs), which form a monophyletic group distinct from the group of flaviviruses that infect vertebrates, comprise KRV, NIEV and cell-fusing agent virus (CFAV), among others [11,13]. Dual-host-affiliated ISFs (dISFs) exemplified by Nounané virus were detected subsequently and group within the mosquito-borne and vertebrate-pathogenic flaviviruses [14].

While ISFs are not known to be pathogenic to vertebrates, they may have an impact on arbovirus infection risk, as some ISFs have been shown to interfere with infection and transmission of mosquito-borne viruses in vitro and in vivo. For example, Nhumirim virus (NHUV) has been shown to reduce ZIKV and DENV viral growth in *Aedes* (*Ae.*) *albopictus* cells [15], and Palm Creek virus (PCV) was shown to reduce WNV transmission in *Culex annulirostris* mosquitoes [16]. Thus, assessing the genetic diversity of ISFs in naturally infected mosquito populations provides a baseline for subsequent studies on their interaction with endemic arboviruses [15,16]. 

Previous studies in Kenya have detected ISFs in mosquitoes. These include KRV in *Ae. mcintoshi*, CFAV in *Ae. aegypti* and Aedes flavivirus in *Ae. luteocephalus* [11,17,18,19]. Despite these previous detections in diverse mosquito species, there is little knowledge on ISFs in blood-fed mosquitoes and just a few have been described in known arbovirus mosquito vectors.

Blood-meal feeding patterns are important epidemiological drivers of pathogen transmission [20]. Certain mosquito species may exhibit preferences for particular hosts: *Ae. aegypti* and *Anopheles gambiae*, for example, preferentially feed on humans [21,22]. Published literature suggests that mosquito blood-feeding patterns can be highly heterogenous [23]. Some mosquito species show opportunistic blood-feeding patterns characterized by mixed feeding on wildlife and humans, possibly serving as bridge vectors that facilitate arbovirus spill-over from sylvatic cycles to humans. In the Afrotropical region, mixed feeding has been reported for *Aedes* mosquitoes including *Ae. africana* and *Ae. Aegypti,* which act as bridge vectors in sylvatic and peridomestic arbovirus transmission cycles [24,25]. Similarly, mixed feeding on humans and wild animals has been reported in *Cx. univittatus* and is associated with WNV transmission at the human–wildlife interface involving migratory birds [19,26,27]. As reported in mosquito–bacterial symbiotic relationships, blood-meals can also alter the gut microbiota of mosquitoes and ticks, depending on the host [28,29]. 

In the present study, we sought to assess ISFs infections in host-seeking and gravid/blood-fed mosquitoes collected in two Kenyan dryland ecosystems: Baringo and Kajiado counties. We also sought to identify the host blood-meal sources of engorged specimens. 

## 2. Materials and Methods

### 2.1. Ethical Approval

Approval of the study (SERU protocol number 3312) was obtained from the Kenya Medical Research Institute, Scientific and Ethics Review Unit (KEMRI-SERU). In addition, prior to each mosquito sampling activity, informed oral consent was sought from local authorities and village elders.

### 2.2. Sampling Sites, Mosquito Surveys and Processing

**Figure 1:**
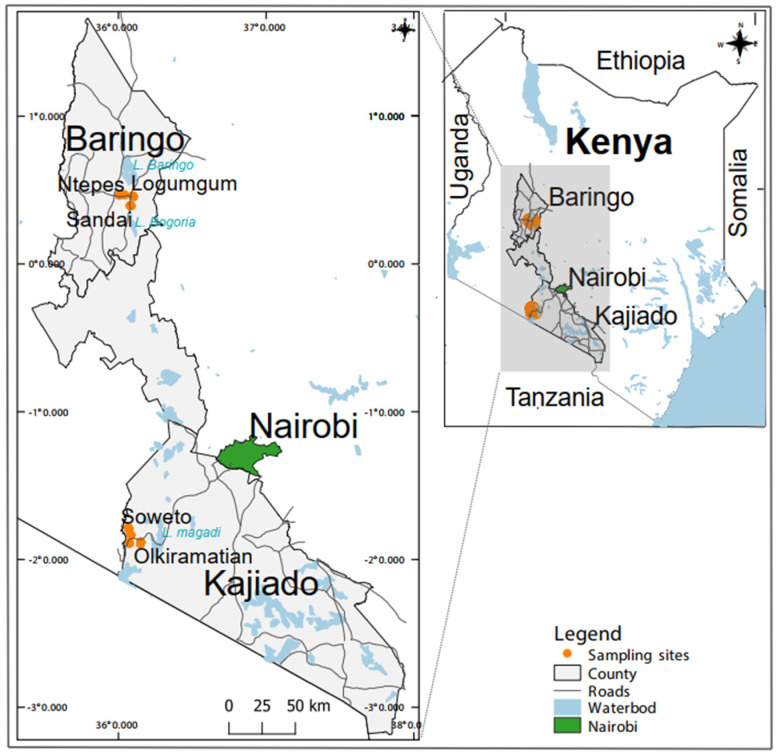
Map showing the five mosquito sampling sites in Baringo and Kajiado counties in Kenya. Two sites in Baringo County (Sandai and Ntepes) have the same agricultural field habitat type and were treated as a single plot. The map was generated in QGIS 2.12 with shape files provided by Natural Earth (http://www.naturalearthdata.com/) and Africa Open data (https://africaopendata.org/dataset/kenya-counties-shapefile) [34].

Mosquitoes were sampled in August 2019 in Baringo County and in July 2020 in Kajiado County just after the rains, when high mosquito activity is expected [30]. Both counties have ecosystems inhabited by diverse livestock, wildlife hosts and humans, and both are associated with arboviral activity [18,31,32,33]. The landscape in both areas is characterized by dry African savannah comprising landscapes dominated by low-lying Acacia bushes, tall grasses and discontinuous trees. Seasonal rivers with low-lying plains provide water for irrigation, grazing land, food for wildlife and livestock, and a source of livelihood for the human population. Kajiado County is located in the southern part of Kenya and shares a border with Tanzania, while the study sites in Baringo County are located ~250 km northwest of Nairobi (Figure 1).

Four plots representing distinct habitat types including agricultural fields (two plots) and wildlife conservation areas (two plots) were identified in each county. In each plot, adult mosquitoes were captured using five traps consisting of one Centers for Disease Control (CDC) Gravid trap targeting gravid mosquitoes, and two CDC light traps model 512 (both John W. Hock Company, Gainesville, FL, USA) and two Biogent (BG) sentinel traps (Biogents AG, Regensburg, Germany) targeting host-questing mosquitoes. All traps were deployed as described previously [35]. In each plot (25 × 25 m), five traps were set 25 m from each other with the CDC Gravid trap in the middle. Plots were set at least 100 m apart from each other. In each sampling site, BG sentinel traps and CDC Gravid traps were set over three days and three nights, respectively, while CDC light traps were set for three nights. The captured mosquitoes were anesthetized with triethylamine and immediately stored in liquid nitrogen in the field and at −80 °C upon arrival at the laboratory at the Martin Lüscher Emerging Infectious Diseases Laboratory (ML-EIDL), *icipe* (International Centre of Insect Physiology and Ecology), Nairobi. Mosquito species were identified using established taxonomic keys [36,37]. For analysis purposes, the identified mosquitoes were pooled by species, sex, abdominal status (unfed/gravid, blood-fed) and trapping location in groups of no more than 25 individuals. Blood-fed mosquitoes were analysed individually. Each individual or mosquito pool was homogenized using a Mini-Beadbeater-16 (Biospec, Bartlesville, OK, USA) in 500−1000 µL of Dulbecco’s phosphate-buffered saline (pH 7.4). The homogenate was centrifuged at 2500 relative centrifugal force (rcf) per minute at 4 °C and stored at −80 °C until further processing.

### 2.3. Blood-meal Source Identification

Genomic DNA was extracted from individual abdominal content of all 80 engorged mosquitoes using DNeasy Blood and Tissue Kit (QIAGEN, Hilden, Germany) following the manufacturer’s protocol. The isolated genomic DNA was used as a template for molecular identification of blood-meal host sources, utilizing PCR-sequencing of the cytochrome c oxidase subunit 1 (cox1) gene fragment, as described earlier [38]. PCR products were purified using ExoSAP-IT (USB Corporation, Cleveland, OH, USA) and submitted for bidirectional Sanger sequencing (Microsynth Seqlab GmbH, Göttingen, Germany). The resulting sequences were compared with reference sequences in GenBank and Barcode of Life (BOLD) databases using BLASTn and BOLD Systems, respectively [39,40].

### 2.4. Detection and Identification of Viruses

To profile flaviviruses associated with mosquitoes, 6665 mosquitoes including 80 blood-fed specimens were tested in 1257 pools (≤25 mosquitoes/pool); the 80 blood-fed mosquitoes were tested individually. Briefly, total RNA was extracted from the supernatant of each homogenate using the Viral RNA Mini Kit (Qiagen, Hilden, Germany) and eluted in a final volume of 80 microliters with nuclease-free water heated to 80 °C. To obtain first-strand complementary DNA (cDNA), the extracted genomic RNA was reverse-transcribed using the Superscript IV Reverse Transcription (RT) kit and random hexamer primers (Invitrogen, Karlsruhe, Germany). Flavivirus infection was confirmed by improved genus-specific RT-PCR using primers Pan-Flavi-F1 (5′-CATTTGGTACATGTGGYT-3′) and Pan-Flavi-R1 (5′-ACAACACMRTCRTCICC-3′) targeting the flavivirus RNA-dependent RNA polymerase (RdRp) gene [41,42]. The amplicons were used for nested PCRs with Pan-Flavi-F2 (5′-CGTAGCWGGMTGGGAYAC-3′) and Pan-Flavi-R2 (5′-CTGTCCTGAICCTCKYTG-3′) primers. PCR products of the expected sizes (580 bp and 230 bp respectively) were determined by 1.5% agarose gel electrophoresis against a molecular weight marker and purified using ExoSAP-IT (USB Corporation, Cleveland, OH, USA). The sequences obtained by Sanger sequencing with forward and reverse primers (Microsynth Seqlab GmbH, Göttingen, Germany) were compared with reference sequences in GenBank using the blast nucleotide function [40].

### 2.5. Virus Isolation and Screening of Cultures

Insect cell lines C6/36 (*Aedes albopictus*) grown in Gibco Leibovitz’s L-15 medium (L-15) supplemented with 5% fetal calf serum (FCS, Gibco, Fisher Scientific) were used for virus isolation. Briefly, 50 μL of homogenate of RT-PCR-positive samples (7 samples) were inoculated onto semi-confluent monolayer cells in a 48-well plate (Nunc, Roskilde, Denmark). Following incubation of infected cells for one hour to allow adsorption, 700 μL of L-15 supplemented with 5% FCS was added to each well. The cells were incubated at 28 °C for seven days and examined daily for cytopathic effects (CPE). Samples suspected to be contaminated by bacteria or fungi present on the mosquitoes were passed through a 0.22 μm syringe filtration, after purification and the addition of a third antibiotic (Nanomycopulitin), isolation was attempted again [43]. The cell culture supernatant was blind-passaged 3 times and the cell culture supernatant of each passage examined for ISFs genome copies by RT-PCR as described above in Section 2.4.

### 2.6. Next-generation Sequencing (NGS)

Viral nucleic acid was extracted from all culture supernatants using NucleoSpin RNA Virus Kit (Macherey-Nagel, Düren, Germany) as described by the manufacturer. First-strand and second-strand cDNA synthesis, DNA library preparation and individual sample purification were carried out using the KAPA HyperPlus kit (Roche Diagnostics, Rotkreuz, Switzerland) following the manufacturer’s protocol. Individual samples were tracked using a tag-sequence incorporated at both ends of the cDNA. The library was deep-sequenced on an Illumina MiSeq next-generation sequencing (NGS) platform and a ~300 bp paired-end read protocol. Bioinformatics analysis, including mapping NGS reads to reference viruses using Geneious Mapper and de novo assembly of identical viral reads using Spades v3.11.1, was carried out as described previously [44]. The resulting consensus sequences were searched against GenBank’s non-redundant protein and nucleotide databases using BLASTx and BLASTn, respectively [40].

### 2.7. Phylogenetic Analysis and Genome Characterization

The partial cox1 sequence dataset generated for blood-meal source identification and the viral RdRp fragment dataset were imported into Geneious Prime [45]. Multiple sequence alignments were performed for each using the MAFFT v. 1.4.0 add-on in Geneious Prime operated under the standard ‘Pairwise/Multiple Align’ option [45,46]. The alignment included the generated sequences in the present study and reference sequences retrieved from GenBank, including CuaCua virus (CuCuV) (GenBank accession no: KX245154) reported in *Mansonia* sp. in Mozambique in 2014 and other flaviviruses. Phylogenetic relationships of the identified viruses and related ISFs were inferred using the maximum likelihood approach in PhyML v. 2.2.4, under the general time reversible (GTR) model of sequence evolution [47]. Branch support was assessed over 1000 bootstrap replicates. The complete coding sequence generated from NGS was annotated in Geneious Prime using InterProScan [45,48] and compared with other mosquito-specific flavivirus genomes.

### 2.8. Statistical Analysis

Differences in the relative abundance of mosquito species at each site were evaluated in R version 4.1.2 [49]. Overall minimum infection rate (MIR) of cISFs was estimated using the PooledInfRate version 4.0 Microsoft Excel Add-In, under the assumption that in every positive pool, only one mosquito is positive [50]. Mosquito blood-feeding pattern was visualized using the bipartite R package [51,52].

### 2.9. Sequence Accession Numbers

The RdRp gene fragments sequences were submitted to GenBank under accession numbers OQ588802–OQ588805 and OQ588808–OQ588810. The Cuacua virus complete coding sequence and raw NGS data were deposited in GenBank and Sequence Read Archive (SRA) under accession numbers OQ588812 and PRJNA995362, respectively.

## 3. Results

### 3.1. Mosquito Composition

A total of 6665 mosquitoes representing 10 genera and 34 species were sampled using CDC light traps, BG sentinel traps and CDC Gravid traps in Baringo and Kajiado counties. More mosquitoes were collected in Kajiado (n = 4336, 65.1%) than in Baringo County (n = 2329, 34.9%). Most of the mosquitoes were captured in CDC light traps (4363, 65.4%), followed by BG sentinel traps (n = 2238, 33.6%) and CDC Gravid traps (n = 64, 1.0%). Overall, there were more female (n = 5236, 78.6%) than male (n = 1429, 21.4%) mosquitoes trapped in Baringo and Kajiado counties. The highest overall collections were registered in Soweto (n = 2659, 39.9%) and Olkiramatian (n = 1677, 25.2%) in Kajiado County, followed by Logumgum in Baringo County (n = 856, 12.8%) (Table 1).

Overall, *Uranotaenia* (*Ur.*) *nigromaculata* (1506, 22.6%), *Mansonia* (*Ma.*) *africana* (1424, 21.4%), *Culex* (*Cx.*) *univittatus* (948, 14.2%) and *Ma. uniformis* (835, 12.5%) dominated the mosquito fauna, representing more than 70% of the collections in terms of relative abundance. The highest mosquito species richness was observed at two sites in Kajiado County, viz. Olkiramatian (24 species) and Soweto (22 species), while Logumgum and Sandai in Baringo County had the lowest species richness with 14 species recorded at each site. The mosquito genus with the highest number of species was *Culex*, with ten species identified, followed by the genera *Anopheles* and *Aedes*, for which seven species were recorded for each. A number of mosquito species, including *Ur. nigromaculata, Ur. pallidoephala*, *Ur. philaroxia*, *Ficalbia uniformis*, *Eretmapodites chrystogastar*, *Cx. zombaensis*, *Cx. tigripes*, *Cx. poicilipes*, *Coquillettidia fuscopenatta*, *Anopheles* (*An.*) *maculipalpis* and *An. coustani*, were exclusive to Kajiado County. Similarly, *An. squamosus*, *Aedes* (*Ae.*) *ethiopicus*, *Ae. metallicus*, *Ae. tricholabis*, *Ae. vittatus*, *Coquillettidia aurites* and *Cx. bitaeniorhynhus* were exclusive to Baringo County. In total, 80 blood-fed mosquitoes were found, mostly originating from Baringo (71.3%, 57/80) with the balance from Kajiado County (28.8%, 23/80). The total number of blood-fed mosquitoes represented 1.2% of the total mosquito catch (80/6665).

### 3.2. Mosquito Blood-Meal Host Sources 

**Figure 2:**
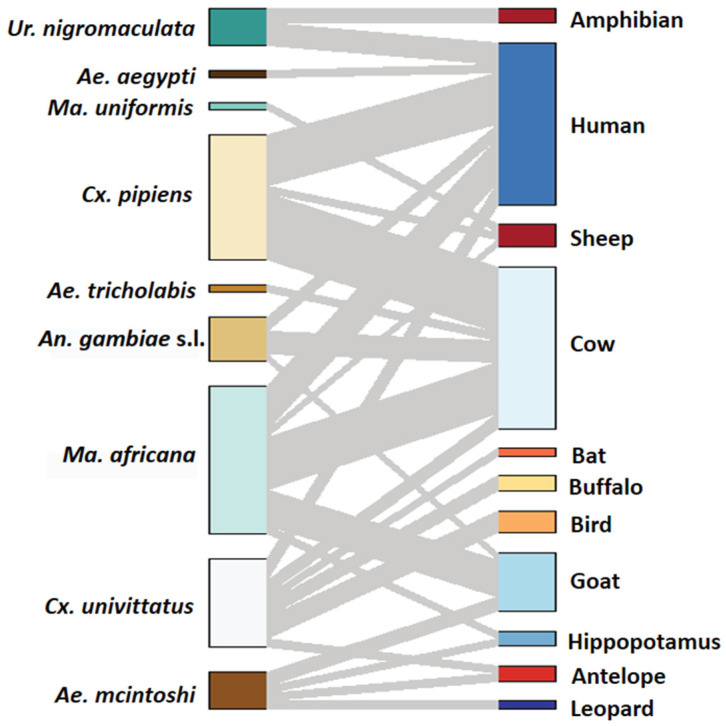
Alluvial diagram showing mosquito blood-feeding patterns. The diagram was visualized using the *bipartite R* package [51,52].

A total of 80 blood-fed mosquitoes comprising nine species were analysed for vertebrate blood-meal sources. In Baringo County, the analyzed samples constituted six species in three sampling sites, while in Kajiado County, the samples constituted six species from two sampling sites. Overall, blood-meal host sources were successfully identified from 68 mosquitoes, representing 85% of the analysed blood-fed samples (n = 80). Eleven different vertebrate blood-meal hosts were identified, including human (*Homo sapiens*) (Hominidae), and six bovid species comprising cattle (*Bos taurus*), goats (*Capra hircus*), sheep (*Ovis aries*), African buffalo (*Syncerus caffer*), hippopotamus (*Hippopotamus amphibius*) and antelope (*Tragelaphus scriptus*) (Figure 2). The other animal blood-meals originated from an amphibian (*Ptychadena mascareniensis*, Ptychadenidae), bird (*Tchagra senegalus*, Malaconotidae), bat (*Epomophorus labiatus*, Epomophorinae) and cat (*Panthera pardus*, Felidae) species (Table 2). *Culex univittatus* had the most diverse blood-meal host sources followed by *Ma. africana* (Table 2). The wildlife conservation site (Olkiramatian) had the greatest variety of detected blood-meal hosts, followed by the agricultural field site (Soweto) in the same county. Further, blood-meal host source diversity was higher in Kajiado (eight vertebrate hosts) than in Baringo County (five vertebrate hosts) (Table 2). Mixed-host feeding on humans and bats, as demonstrated by a double sequencing signal observed in sequence chromatograms, was recovered in a single specimen of *Cx. univittatus* in Soweto, Kajiado County. Overall, cattle (28.4%, n = 23) was the most frequently detected vertebrate blood-meal source followed by humans (27.2%, n = 22) and goats (9.9%, n = 8). 

### 3.3. Flavivirus Sequence Detections in Mosquitoes 

**Figure 3:**
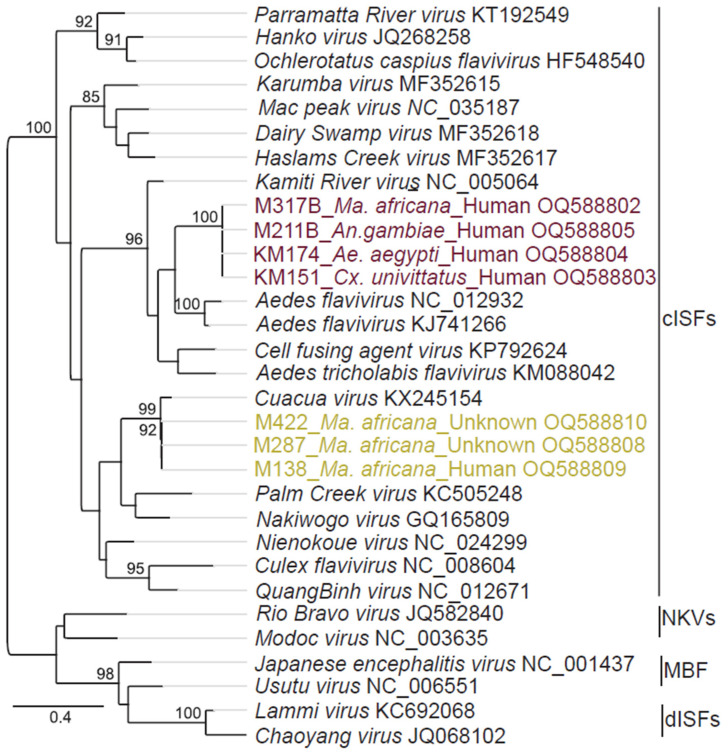
Maximum likelihood phylogenetic tree reconstructed using partial 500 bp-RNA-dependent RNA polymerase gene of representative flaviviruses and viral sequences detected in the present study. The phylogenetic tree was reconstructed with PhyML v. 2.2.4 employing GTR substitution model. Bootstrap values of 70% and above are shown. The flaviviruses groups are indicated: cISFs: classical insect-specific flaviviruses; MBF: mosquito-borne flaviviruses; NKV: no-known-vector flaviviruses; dISFs: dual-host affiliated ISFs. The viruses of the same species are shown in the same colour. Mosquito species referenced as either human or unknown represent blood-meal sources of engorged (blood-fed) specimens while unfed represent specimens that had not taken a blood meal.

All mosquito pools including blood-fed specimen were tested for infections with flaviviruses by generic RT-PCR; identifying seven *Flavivirus* sequence fragments (Table 3). Overall, *Flavivirus* infection rate was 0.1% (7/1257, 95% confidence interval ((CI) 0.0–0.2), including the detections in blood-fed mosquitoes. Five of the positive samples were mosquitoes that had fed on humans, including *Ae. aegypti*, *An. gambiae* s.l., *Ma. africana* and *Cx. univittatus.* The sequences of four samples (KM174, KM151, M211B and M317B) were identical to each other across the RdRp gene region sequenced (Figure S1) and showed 72% nucleotide (nt) identity to the cISF Aedes flavivirus (GenBank accession no: KJ741266) detected in *Ae. albopictus* collected in Thailand in 2014 [12,53]. Three further sequences, detected in three blood-fed mosquitoes (M138, M422 and M287), showed 99–100% nt identity among each other and 94% nt identity to CuaCua virus (CuCuV) (GenBank accession no: KX245154) reported in *Mansonia* sp. in Mozambique, in 2014. All three CuCuV detections were in Baringo County, two in Ntepes and one in Logumgum. Phylogenetic analyses confirmed that the sequences of the samples KM174, KM151, M211B and M317B clustered as a sister taxon to Aedes flavivirus and that the sequences of the samples M138, M422 and M287 clustered in a clade with CuCuV, both with significant bootstrap support (Figure 3).

### 3.4. Virus Isolation and Genome Organization 

All virus-positive mosquito homogenates were used for virus isolation attempts in cell culture. Viral genome copies were detected in cell culture supernatants after two blind passages for the flavivirus sequences (KM151, KM174 and M211B) as well as for the CuCuV-containing pool M138, albeit without prominent cytopathic effects (CPEs). Cell culture supernatants of passage three of the four samples were negative for viral genome copies. In addition, viral genome copies were not detected in cell culture supernatants of the three samples including M287, M422 and 317B in all three passages (passages one, two and three). Further phenotypic virus investigations such as growth analyses were not explored as part of this study.

**Figure 4:**
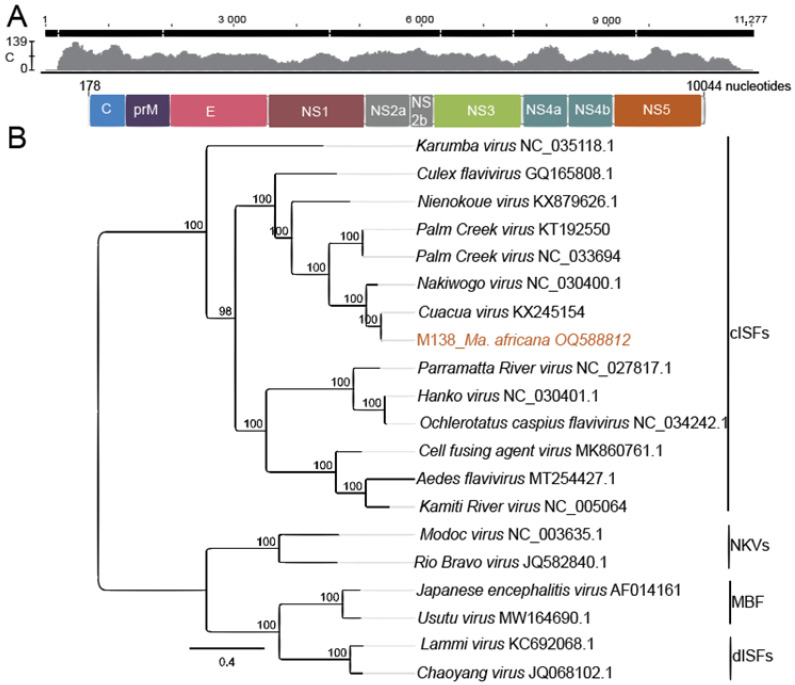
Cuacua virus schematic genome organisation and maximum likelihood phylogenetic tree. (**A**), Schematic genome organisation. Predicted structural and non-structural proteins are indicated. The distribution and number of obtained NGS reads are shown in the graph above the genome segment, with the *y*-axis (c: coverage) showing the maximum number of overlapping reads in every genome position; (**B**), Maximum likelihood phylogenetic tree of complete coding sequences. The flavivirus groups are indicated: cISFs: classical insect-specific flaviviruses; MBF: mosquito-borne flaviviruses; NKV: no-known-vector flaviviruses; dISFs: dual-host affiliated ISFs. The phylogenetic tree was reconstructed with PhyML v. 2.2.4 employing an LC substitution model. CuCuV sequences identified in this study are shown in brown.

All PCR-positive cell culture supernatants were subjected to NGS for genome sequencing. However, these attempts were not successful for six of the seven samples infected with CuCuV and the potential novel ISF. The coding-complete genome of CuCuV M138 was successfully sequenced. Homologous with other flavivirus genomes, the genome of M138 comprised a single-stranded ORF of 10,044 nt in length with a G + C content of 46.6% and encoding a 3272 amino acid viral polyprotein (seven non-structural proteins and three structural proteins) (Figure 4A). Genome sequence comparison against GenBank’s non-redundant nucleotide database confirmed a 95.8% identity to CuCuV (KX245154) detected in *Mansonia* sp. from Mozambique [12]. Compared with the published sequence, 66 and 603 additional nucleotides were recovered on the 5′ and 3′ ends, respectively. The amino acid identity at the deduced polyprotein level between the identified CuCuV (M138) and the Mozambique strain was 99%. Maximum likelihood phylogenomic analyses further showed that the detected virus grouped with CuCuV detected in mosquitoes from Mozambique, forming a robust monophyletic clade (100% clade probability) (Figure 4B) [12]. 

## 4. Discussion

Among the 34 mosquito species recorded from five sampling sites were *Ae. aegypti*, *Ae. mcintoshi*, *Ae. simpsoni*, *Cx. univittatus* and *Ma. africana*, which are known vectors of YFV, DENV and Rift Valley fever virus (RVFV) [25,54,55]. Mosquitoes were more abundant in Kajiado County (particularly in Soweto) than in Baringo County. Mosquito species distribution and abundance varied by habitat type and sampling site. For instance, mosquito abundance including *Ur. nigromaculata* was particularly high in Soweto, likely attributed to the availability of many breeding sites created by the forested area and extensive irrigation activities in agricultural fields where trapping was conducted [56]. Additionally, irrigated areas as observed in Soweto and Ntepes have been shown to harbour high densities of mosquitoes such as *Culex* spp. and *Mansonia* spp., explaining the abundance of *Cx. annulioris* and *Cx. cinereus* in Soweto and *Ma. africana* in Ntepes [56]. *Ma. africana* and *Ma. uniformis* dominated mosquito species collection in Logumgum, suggesting that the swamp and the marshland vegetation in the area provided a suitable breeding site for the vector, as described earlier [56]. The economic activities in the region, including subsistence farming involving extensive irrigation activities and wild animal conservancies, provide breeding sites for mosquitoes, while the frequent movement of herders in search of pasture and water within the region could increase the contact between human and sylvatic mosquitoes [57]. To the best of our knowledge, this study provides the first report on mosquito diversity, blood-feeding pattern and abundance in Kajiado County (Kajiado North constituency).

Our trapping tools targeted host-seeking and gravid mosquitoes while poorly trapping blood-fed mosquitoes. Nonetheless, our blood-meal data showed feeding on 11 vertebrate hosts, including humans, cattle, goats, sheep, buffalos, hippopotamus, antelopes, amphibians, birds, bats and leopards, in agreement with previous studies reporting diverse hosts for mosquito species [21,58]. The apparent high feeding on human and cattle hosts could be ascribed to their abundance in the areas compared to other vertebrate animals. All mosquitoes demonstrated mammophilic blood-feeding habits except *Ur. nigromaculata* and *Cx. univittatus*, which had amphibians and birds as additional blood-meal sources. Ornithophilic tendencies and reports of feeding on humans, sheep and dogs are widely documented in *Cx. univittatus* [26,59]. There were more diverse blood-meal host sources in Kajiado County (eight vertebrate hosts) than in Baringo County (five vertebrate hosts). Although there was no vertebrate host census data for the study area, the disparity in the diversity of blood-meal hosts in the two study areas could be attributable to mosquito preference for certain hosts and/or variable vertebrate host densities. At each sampling site, there appeared to be an association between available vertebrate hosts and the blood-meal source detected. For example, at Logumgum there is a swamp inhabited by hippopotamus, explaining its detection in the meals of the flood-water mosquito *Ae. mcintoshi* and *Ma. africana*. Similarly, the blood meals of mosquitoes sampled from the wildlife conservancy at Olkiramatian reflected the wild animals present, viz. *Ae. mcintoshi* (leopard) and *Cx. univittatus* (buffalo). The mosquito species that feed on wildlife and also feed on humans potentially serve as bridge vectors that facilitate arbovirus spill-over from sylvatic cycles to humans [60]. An extended study of blood-fed mosquitoes would provide a more accurate view of the mosquitoes’ blood-feeding pattern. 

Cuacua virus (CuCuV) is a cISF initially detected in a pool of *Mansonia* sp. collected in 2014 from Zambezi province, Mozambique [12]. Here, we report its first detection in *Ma. africana* with evidence of human blood-feeding, in the dryland ecosystem of Baringo County in Kenya, and the generation of a coding-complete genome sequence using NGS. Similar to its previous association with *Mansonia* mosquitoes [12], the identification of CuCuV in three individual blood-fed *Ma. africana* specimens, including one that had a human blood-meal (two blood-meal sources were unknown), suggests that *Mansonia* mosquitoes are infected by CuCuV over a wider geographic area. Another undescribed potential cISF in a sister-relationship to Aedes flavivirus KJ741266 with 72% nucleotide sequence identity to this reference strain was detected in four individual blood-fed mosquitoes, viz. *Ae. aegypti*, *An. gambiae* s.l., *Ma. africana* and *Cx. univittatus*, that all had human blood-meals. Whilst a near full-length genome sequence was generated for the CuCuV detected in *Ma. africana*, a shortcoming of this study was the inability to generate reference genome sequences for the other potential novel flavivirus lineage, possibly due to the lack of an isolate. Despite this limitation, this study has highlighted high levels of flavivirus diversity in mosquitoes from two under-studied regions of Kenya.

The identification of cISFs is not new in Kenya; however, earlier detections were largely made in unfed mosquitoes, unlike the present study in which five of the seven flavivirus-positive mosquitoes were found to have human-derived blood-meals and only two were detected in mosquitoes with unknown blood-meal sources [18,19,61,62]. Classical ISFs have previously been detected in *An. gambiae* s.l., agreeing with the present detection of flavivirus in *An. gambiae* s.l. [63]. Additional detections in *Cx. univittatus* and *Ae. aegypti* suggest a wider presence and interactions with diverse mosquito species. Whilst we were unable to obtain any cISFs isolates as depicted by the absence of viral genome copies beyond passage two and in seven samples, the findings highlight CuCuV presence in East Africa and expand knowledge on the diversity of ISFs. 

The detection of identical short cISFs RdRp gene sequences with 100% nucleotide identity in four mosquito species including *Cx. univittatus*, *Ae. aegypti*, *An. gambiae* s.l. and *Ma. africana* supports the suggestion of inter-species/genera cISF horizontal transmission in mosquitoes made by Guégan et al. [64] and requires further investigation. In contrast, vertical cISF transmission, which has been demonstrated both experimentally and in natural ecologies, is well established [17,18,65]. While we detected the flaviviruses in blood-fed mosquitoes, the results raise an unaddressed question as to why cISFs were preferentially detected in mosquitoes with human-derived blood meals. In *Ae. aegypti* and *Ae. albopictus*, hsa-miR-150-5p microRNAs in human blood has been shown to facilitate flavivirus infection [66]. In addition, human blood proteins could offer more protection from degradation to the virus or viral RNA than other blood-meal sources; however, this remains to be established. The content of blood such as hemoglobin (HB), hematocrit (HCT) and total protein (TP) varies in different hosts [67], which could influence the differential proliferation of cISFs as noted for midgut bacterial symbionts in mosquitoes [29]. Further, catabolism of blood meals results in the generation of reactive oxygen species, which may induce changes in gut bacterial composition differently by blood-host types [68,69]. We note that the reason for the observation remains speculative and warrants additional research to confirm the findings. Nonetheless, this is the first report describing an association of cISFs in mosquitoes with a background of human-derived blood in the region. Further experiments are necessary to investigate modulation of cISF prevalence in mosquitoes by blood-meal host type. If found to impact the transmission of cISFs, then their differential presence could be a determining factor of variation in vector competence among vectors at individual and population levels. 

## 5. Conclusions

We detected ISFs and CuCuV in the main vector species of YFV, DENV and RVFV, including *Ae. aegypti*, *Ma. africana* and *Cx. univittatus* with human-derived blood meals. Mosquito infections with ISFs could confer a benefit, as they may reduce arbovirus transmission and, thus, the risk of disease. However, as the detected viruses have so far not been studied in co-infection experiments, it is unknown if they influence arbovirus replication and transmission. While blood-meal analysis revealed diverse vertebrate hosts for mosquito species, opportunistic blood-feeding involving wildlife and human was also observed as in the case of *Cx. univittatus* mixed-blood-feeding on humans and bats. Our findings confirm CuCuV presence in East Africa and expand knowledge on the extensive diversity of cISFs. Further studies are needed to explore the role of host blood-meal and cISFs on arbovirus transmission dynamics. 

## Figures and Tables

**Table 1:** Mosquito abundance at three Baringo County sampling sites and two Kajiado County sampling sites in Kenya.

	Baringo County									Kajiado County						
	Ntepes			Sandai			Logumgum			Soweto			Olkiramatian			n (%)
**Sampling method**	BG	CDC-LT	CDC-Gravid	BG	CDC-LT	CDC-Gravid	BG	CDC-LT	CDC-Gravid	BG	CDC-LT	CDC-Gravid	BG	CDC-LT	CDC-Gravid	
	370	313	35	479	257	19	395	454	7	356	2303	0	638	1036	3	6665
**Sex**																
Female	364	310	31	476	254	18	394	446	4	316	1065	0	620	935	3	5236 (78.6)
Male	6	3	4	3	3	1	1	8	3	40	1238	0	18	101	0	1429 (21.4)
**Mosquito species**																
*Uranotaenia nigromaculata*	0	0	0	0	0	0	0	0	0	57	1387	0	0	62	0	1506 (22.6)
*Mansonia africana*	246	129	0	98	24	0	247	346	5	1	0	0	177	151	0	1424 (21.4)
*Culex univittatus*	0	16	0	5	2	0	0	0	0	97	255	0	260	313	0	948 (14.2)
*Mansonia uniformis*	50	14	0	325	202	15	122	69	0	0	2	0	17	19	0	835 (12.5)
*Anopheles funestus* s.l.	0	0	0	2	0	0	0	0	0	40	157	0	37	89	0	325 (4.9)
*Culex vansomereni*	3	3	0	0	3	0	0	6	0	19	22	0	53	156	0	265 (4.0)
*Culex pipiens*	7	64	5	15	8	1	4	1	0	16	35	0	46	58	0	260 (3.9)
*Culex annulioris*	0	0	0	0	1	0	0	0	0	86	152	0	0	19	0	258 (3.9)
*Culex cinereus*	0	0	0	0	0	0	0	0	0	1	102	0	0	4	0	107 (1.6)
*Aedes aegypti*	29	0	23	20	1	2	0	1	0	3	0	0	17	0	0	96 (1.4)
*Aedes mcintoshi*	4	5	0	1	0	0	4	0	0	4	24	0	7	43	0	92 (1.4)
*Anopheles coustani*	0	0	0	0	0	0	0	0	0	7	31	0	5	25	0	68 (1.0)
*Anopheles gambiae* s.l.	4	24	4	0	1	0	1	2	1	4	4	0	6	15	0	66 (1.0)
*Ficalbia mediolineata*	0	0	0	0	0	0	0	0	0	11	35	0	0	12	0	58 (0.9)
*Ficalbia splendens*	0	0	0	0	0	0	0	2	0	0	28	0	0	27	0	57 (0.9)
*Mansonia* spp.	3	12	0	0	0	0	11	5	0	0	0	0	3	2	0	36 (0.5)
*Aedes tricholabis*	13	8	0	0	0	0	0	2	0	0	0	0	0	0	0	23 (0.3)
*Aedes vittatus*	1	10	0	0	0	0	4	5	1	0	0	0	0	0	0	21 (0.3)
*Anopheles* spp.	0	2	0	1	0	0	0	0	0	0	7	0	5	5	0	20 (0.3)
*Culex ethiopicus*	0	0	0	2	7	0	0	1	0	4	5	0	0	1	0	20 (0.3)
*Orthopodomyia* spp.	1	14	1	0	0	0	0	2	0	0	0	0	0	0	0	18 (0.3)
*Uranotaenia philaroxia*	0	0	0	0	0	0	0	0	0	1	16	0	0	0	0	17 (0.3)
*Uranotaenia* spp.	0	0	0	0	0	0	0	0	0	0	15	0	0	2	0	17 (0.3)
*Culex* spp.	0	0	1	0	2	0	0	1	0	0	2	0	1	7	3	17 (0.3)
*Culex tigriepes*	0	0	0	0	0	0	0	0	0	2	9	0	1	4	0	16 (0.2)
*Aedes simpsoni* s.l.	5	0	0	7	0	0	2	0	0	1	0	0	0	0	0	15 (0.2)
*Anopheles pharoensis*	0	5	0	1	2	0	0	0	0	0	0	0	1	4	0	13 (0.2)
*Culex poicilipes*	0	0	0	0	0	0	0	0	0	0	6	0	1	5	0	12 (0.2)
*Aedes* spp.	1	3	1	2	0	1	0	0	0	1	1	0	0	0	0	10 (0.2)
*Anopheles maculipalpis*	0	0	0	0	0	0	0	0	0	0	8	0	0	0	0	8 (0.1)
*Culex bitaeniorhynhus*	0	0	0	0	0	0	0	7	0	0	0	0	0	0	0	7 (0.1)
*Ficalbia* spp.	0	1	0	0	0	0	0	0	0	0	0	0	0	6	0	7 (0.1)
*Aedes ethiopicus*	0	1	0	0	0	0	0	4	0	0	0	0	0	0	0	5 (0.1)
*Aedes metallicus*	3	0	0	0	1	0	0	0	0	0	0	0	0	0	0	4 (0.1)
*Eretmapodite* spp.	0	0	0	0	3	0	0	0	0	0	0	0	0	0	0	3 (0.0)
*Ficalbia uniformis*	0	0	0	0	0	0	0	0	0	0	0	0	0	3	0	3 (0.0)
*Uranotaenia pallidoephala*	0	0	0	0	0	0	0	0	0	0	0	0	0	3	0	3 (0.0)
*Anopheles* *squamosus*	0	1	0	0	0	0	0	0	0	0	0	0	0	0	0	1 (0.0)
*Coquillettidia aurites*	0	1	0	0	0	0	0	0	0	0	0	0	0	0	0	1 (0.0)
*Coquillettidia fuscopenatta*	0	0	0	0	0	0	0	0	0	0	0	0	0	1	0	1 (0.0)
*Culex zombaensis*	0	0	0	0	0	0	0	0	0	1	0	0	0	0	0	1 (0.0)
*Eretmapodite chrystogastar*	0	0	0	0	0	0	0	0	0	0	0	0	1	0	0	1 (0.0)
**n**	**370**	**313**	**35**	**479**	**257**	**19**	**395**	**454**	**7**	**356**	**2303**	**0**	**638**	**1036**	**3**	**6665**
**Blood-fed**	12	34	0	1	0	0	3	6	1	3	7	0	5	8	0	**80 (1.2)**

n: number of mosquitoes collected; CDC-Gravid: Centers for Disease Control gravid trap; CDC-LT: Centers for Disease Control light trap two CDC light trap; BG: Biogent sentinel trap.

**Table 2:** Blood-meal sources of individual mosquitoes sampled from Baringo and Kajiado Counties, Kenya.

County	Study Area	Species	n	Human	Cattle	Goat	Sheep	Amphibian	Bird	Antelope	Leopard	Buffalo	Hippopotamus	Bat	UN
Baringo	Ntepes	*Culex pipiens*	20	6	9	0	1	0	0	0	0	0	0	0	4
		*Mansonia africana*	17	3	4	5	1	0	0	0	0	0	0	0	4
		*Anopheles gambiae* s.l.	5	2	2	1	0	0	0	0	0	0	0	0	0
		*Aedes mcintoshi*	2	0	0	2	0	0	0	0	0	0	0	0	0
		*Aedes tricholabis*	1	0	1	0	0	0	0	0	0	0	0	0	0
		*Mansonia uniformis*	1	0	0	0	1	0	0	0	0	0	0	0	0
	Logumgum	*Mansonia africana*	8	3	2	0	0	0	0	0	0	0	1	0	2
		*Ae. mcintoshi*	1	0	0	0	0	0	0	0	0	0	1	0	0
		*Anopheles gambiae* s.l.	1	0	1	0	0	0	0	0	0	0	0	0	0
	Sandai	*Mansonia africana*	1	0	1	0	0	0	0	0	0	0	0	0	0
		**n (%)**	**57 (71.3)**	**14 (17.5)**	**20 (25.0)**	**8 (10.0)**	**3 (3.8)**	**0**	**0**	**0**	**0**	**0**	**2 (2.5)**	**0**	**10 (12.5)**
Kajiado	Olkiramatian	*Culex* *univittatus*	6	1	2	0	0	0	1	0	0	2	0	0	0
		*Culex* sp.	2	0	1	0	0	0	0	0	0	0	0	0	1
		*Aedes mcintoshi*	2	0	0	0	0	0	0	1	1	0	0	0	0
		*Culex* *pipiens*	1	1	0	0	0	0	0	0	0	0	0	0	0
		*Mansonia africana*	1	0	0	0	0	0	0	0	0	0	0	0	1
	Soweto	*Culex univittatus*	5	2 *	0	0	0	0	2	1	0	0	0	1 *	0
		*Uranotaenia nigromaculata*	5	3	0	0	0	2	0	0	0	0	0	0	0
		*Aedes aegypti*	1	1	0	0	0	0	0	0	0	0	0	0	0
		**n (%)**	**23 (28.8)**	8 (10.0) *	**3 (3.8)**	**0**	**0**	**2 (2.5)**	**3 (3.8)**	**2 (2.5)**	**1 (1.3)**	**2 (2.5)**	**0**	**1 (1.3)**	**2 (2.5)**
**n (%)**			**80**	**22 (27.2)**	**23 (28.4)**	**8 (9.9)**	**3 (3.8)**	**2 (2.5)**	**3 (3.** **8)**	**2 (2.5)**	**1 (1.3)**	**2 (2.5)**	**2 (2.5)**	**1 (1.3)**	**12 (14.8)**

n: number of mosquitoes; UN: Unidentified blood-meals; * mixed-bloodmeals.

**Table 3:** Description of flavivirus positive samples.

Sample Code	Mosquito Species	No of Mosquitoes	Blood-Meal Source/ Abdomen Status	Closely Related Flavivirus Species	% ID (nt)	Sequence Length (bp)	Genbank Acession No.
M138	*Mansonia africana*	1	Human	CuaCua virus	94	218	OQ588809
M287	*Mansonia africana*	1	Unknown	CuaCua virus	94	201	OQ588808
M422	*Mansonia africana*	1	Unknown	CuaCua virus	94	201	OQ588810
M211B	*Anopheles gambiae* s.l.	1	Human	Aedes flavivirus	72	211	OQ588805
M317B	*Mansonia africana*	1	Human	Aedes flavivirus	72	212	OQ588802
KM151	*Culex univittatus*	1	Human	Aedes flavivirus	72	212	OQ588803
KM174	*Aedes aegypti*	1	Human	Aedes flavivirus	72	219	OQ588804

No: number; ID: identity; nt: nucleotides.

## Data Availability

Sequences generated were deposited to GenBank under accession numbers OQ588802–OQ588805, OQ588808–OQ588810 and OQ588812, and Sequence Read Archive (SRA) under the accession number PRJNA995362. Other data presented in the study are available in the article and as supplements.

## Supplementary Materials

The following supporting information can be downloaded at: https://www.mdpi.com/article/10.3390/pathogens12070967/s1, Figure S1: Classical insect-specific flaviviruses (cISFs) distance matrix.
