# Supplementary material for: Analyses of Mosquito Species Composition, Blood-Feeding Habits and Infection with Insect-Specific Flaviviruses in Two Arid, Pastoralist-Dominated Counties in Kenya
Source: Pathogens. 2023 Jul 24;12(7):967. doi: 10.3390/pathogens12070967 (PMC10386387; doi:10.3390/pathogens12070967)
Supplement: Supplementary file 1 [file pathogens-12-00967-s001.zip › pathogens-2301831-supplementary.pdf]

	M422	M287	M138	M317B	M211B	KM151	KM174	Aedes flavivirus NC_012932	Aedes flavivirus KJ741266	Cell fusing agent virus KP792624	Aedes tricholabis flavivirus KM088042	Kamiti River virus NC_005064	Mac peak virus NC_035187	Karumba virus MF352615	Dairy Swamp virus MF352618	Haslams Creek virus MF352617	Parramatta River virus KT192549	Hanko virus JQ268258	Ochlerotatus caspius flavivirus HF548540	Palm Creek virus KC505248	Nakiwogo virus GQ165809	Cuacua virus KX245154	Nienokoue virus NC_024299	Culex flavivirus NC_008604	QuangBinh virus NC_012671	Rio Bravo virus JQ582840	Modoc virus NC_003635	Japanese encephalitis virus NC_001437	Usutu virus NC_006551	Chaoyang virus JQ068102	Lammi virus KC692068
M422		99%	99%	55%	55%	55%	55%	59%	59%	58%	56%	61%	59%	63%	63%	62%	59%	56%	59%	70%	72%	94%	70%	60%	64%	49%	48%	44%	45%	46%	49%
M287	99%		100%	55%	55%	55%	55%	59%	59%	59%	56%	62%	60%	63%	64%	63%	59%	56%	59%	70%	72%	94%	70%	60%	64%	50%	49%	44%	45%	47%	49%
M138	99%	100%		55%	55%	55%	55%	59%	59%	58%	56%	62%	59%	63%	64%	63%	60%	56%	59%	70%	72%	95%	70%	60%	64%	50%	49%	44%	45%	47%	49%
M317B	55%	55%	55%		100%	100%	100%	72%	72%	67%	67%	75%	54%	55%	60%	54%	56%	58%	58%	59%	63%	54%	57%	51%	55%	44%	45%	40%	43%	47%	43%
M211B	55%	55%	55%	100%		100%	100%	72%	72%	67%	67%	75%	54%	55%	60%	54%	56%	58%	58%	59%	63%	54%	57%	51%	55%	44%	45%	40%	43%	47%	43%
KM151	55%	55%	55%	100%	100%		100%	72%	72%	67%	67%	75%	54%	55%	60%	54%	56%	58%	58%	59%	63%	54%	57%	51%	55%	44%	45%	40%	43%	47%	43%
KM174	55%	55%	55%	100%	100%	100%		72%	72%	67%	67%	75%	54%	55%	60%	54%	56%	58%	58%	59%	63%	54%	57%	51%	55%	44%	45%	40%	43%	47%	43%
Aedes flavivirus NC_012932	59%	59%	59%	72%	72%	72%	72%		91%	70%	68%	71%	59%	57%	59%	60%	59%	61%	62%	61%	62%	56%	55%	55%	56%	44%	47%	42%	43%	46%	46%
Aedes flavivirus KJ741266	59%	59%	59%	72%	72%	72%	72%	91%		70%	71%	74%	59%	57%	61%	61%	56%	60%	60%	62%	62%	57%	58%	51%	57%	44%	47%	43%	45%	45%	44%
Cell fusing agent virus KP792624	58%	59%	58%	67%	67%	67%	67%	70%	70%		74%	73%	60%	59%	59%	59%	63%	59%	59%	59%	65%	56%	59%	56%	53%	44%	46%	44%	43%	47%	45%
Aedes tricholabis flavivirus KM088042	56%	56%	56%	67%	67%	67%	67%	68%	71%	74%		75%	57%	60%	59%	60%	64%	61%	61%	60%	60%	56%	61%	52%	55%	42%	44%	44%	43%	43%	44%
Kamiti River virus NC_005064	61%	62%	62%	75%	75%	75%	75%	71%	74%	73%	75%		59%	61%	62%	60%	61%	58%	59%	65%	64%	60%	62%	55%	59%	46%	43%	46%	48%	47%	45%
Mac peak virus NC_035187	59%	60%	59%	54%	54%	54%	54%	59%	59%	60%	57%	59%		75%	76%	76%	63%	63%	65%	58%	60%	59%	63%	64%	58%	49%	47%	47%	46%	47%	47%
Karumba virus MF352615	63%	63%	63%	55%	55%	55%	55%	57%	57%	59%	60%	61%	75%		74%	75%	64%	61%	62%	59%	63%	64%	68%	63%	64%	49%	45%	48%	43%	42%	46%
Dairy Swamp virus MF352618	63%	64%	64%	60%	60%	60%	60%	59%	61%	59%	59%	62%	76%	74%		81%	62%	60%	63%	57%	63%	61%	67%	57%	58%	48%	46%	44%	44%	42%	43%
Haslams Creek virus MF352617	62%	63%	63%	54%	54%	54%	54%	60%	61%	59%	60%	60%	76%	75%	81%		62%	62%	62%	60%	64%	62%	67%	61%	59%	47%	48%	46%	42%	42%	43%
Parramatta River virus KT192549	59%	59%	60%	56%	56%	56%	56%	59%	56%	63%	64%	61%	63%	64%	62%	62%		75%	77%	57%	56%	57%	62%	61%	54%	48%	49%	46%	46%	43%	48%
Hanko virus JQ268258	56%	56%	56%	58%	58%	58%	58%	61%	60%	59%	61%	58%	63%	61%	60%	62%	75%		89%	62%	57%	54%	59%	56%	57%	51%	50%	45%	49%	49%	47%
Ochlerotatus caspius flavivirus HF548540	59%	59%	59%	58%	58%	58%	58%	62%	60%	59%	61%	59%	65%	62%	63%	62%	77%	89%		55%	56%	56%	57%	55%	56%	49%	50%	44%	47%	49%	48%
Palm Creek virus KC505248	70%	70%	70%	59%	59%	59%	59%	61%	62%	59%	60%	65%	58%	59%	57%	60%	57%	62%	55%		72%	69%	66%	61%	63%	50%	48%	43%	45%	47%	46%
Nakiwogo virus GQ165809	72%	72%	72%	63%	63%	63%	63%	62%	62%	65%	60%	64%	60%	63%	63%	64%	56%	57%	56%	72%		70%	68%	63%	65%	51%	49%	44%	45%	45%	49%
Cuacua virus KX245154	94%	94%	95%	54%	54%	54%	54%	56%	57%	56%	56%	60%	59%	64%	61%	62%	57%	54%	56%	69%	70%		69%	60%	64%	51%	48%	44%	43%	46%	48%
Nienokoue virus NC_024299	70%	70%	70%	57%	57%	57%	57%	55%	58%	59%	61%	62%	63%	68%	67%	67%	62%	59%	57%	66%	68%	69%		62%	69%	50%	49%	48%	45%	44%	46%
Culex flavivirus NC_008604	60%	60%	60%	51%	51%	51%	51%	55%	51%	56%	52%	55%	64%	63%	57%	61%	61%	56%	55%	61%	63%	60%	62%		70%	49%	42%	43%	44%	46%	44%
QuangBinh virus NC_012671	64%	64%	64%	55%	55%	55%	55%	56%	57%	53%	55%	59%	58%	64%	58%	59%	54%	57%	56%	63%	65%	64%	69%	70%		53%	46%	42%	43%	44%	44%
Rio Bravo virus JQ582840	49%	50%	50%	44%	44%	44%	44%	44%	44%	44%	42%	46%	49%	49%	48%	47%	48%	51%	49%	50%	51%	51%	50%	49%	53%		66%	56%	61%	51%	52%
Modoc virus NC_003635	48%	49%	49%	45%	45%	45%	45%	47%	47%	46%	44%	43%	47%	45%	46%	48%	49%	50%	50%	48%	49%	48%	49%	42%	46%	66%		61%	57%	53%	57%
Japanese encephalitis virus NC_001437	44%	44%	44%	40%	40%	40%	40%	42%	43%	44%	44%	46%	47%	48%	44%	46%	46%	45%	44%	43%	44%	44%	48%	43%	42%	56%	61%		73%	57%	65%
Usutu virus NC_006551	45%	45%	45%	43%	43%	43%	43%	43%	45%	43%	43%	48%	46%	43%	44%	42%	46%	49%	47%	45%	45%	43%	45%	44%	43%	61%	57%	73%		64%	64%
Chaoyang virus JQ068102	46%	47%	47%	47%	47%	47%	47%	46%	45%	47%	43%	47%	47%	42%	42%	42%	43%	49%	49%	47%	45%	46%	44%	46%	44%	51%	53%	57%	64%		82%
Lammi virus KC692068	49%	49%	49%	43%	43%	43%	43%	46%	44%	45%	44%	45%	47%	46%	43%	43%	48%	47%	48%	46%	49%	48%	46%	44%	44%	52%	57%	65%	64%	82%	

Figure S1: Classical Insect specific flaviviruses (cISFs) distance matrix. Distance matrix of the partial RdRp gene showing of representative viruses and samples sequenced in the present study (red). Light grey to dark shades represent nucleotide distances.
